# Services and interventions needed to prevent secondary health conditions throughout the life span of people with spinal cord injury, South Africa

**DOI:** 10.4102/ajod.v11i0.881

**Published:** 2022-11-11

**Authors:** Sonti I. Pilusa, Hellen Myezwa, Joanne Potterton

**Affiliations:** 1 Department of Physiotherapy, Faculty of Therapeutic Sciences, University of the Witwatersrand, Johannesburg, South Africa; 2 Faculty of Therapeutic Sciences, University of the Witwatersrand, Johannesburg, South Africa

**Keywords:** spinal cord injury, model of care, prevention, secondary health conditions, secondary complications

## Abstract

**Background:**

Current evidence suggests a need for a care model that supports the prevention of secondary health conditions in people with spinal cord injury. Multiple complex factors influence the prevention of secondary health conditions. There is a need for holistic and systems-based prevention approaches, which target multiple levels.

**Objective:**

To identify the services and interventions needed to prevent secondary health conditions throughout the life span of people with spinal cord injury.

**Method:**

We used a descriptive qualitative approach. Data was collected using focus group discussions with professionals in the rehabilitation field. The recorded group discussions were transcribed verbatim, and content analysis was conducted.

**Results:**

Four focus group discussions were conducted. Four themes emerged from the analyses: patient-centred care, access to resources, promotion of health, and skilled healthcare workers.

**Conclusions:**

The suggested services and interventions needed to prevent secondary health conditions target the individuals with spinal cord injury (SCI), health providers, health systems care approach and other sectors outside the health system. These services and interventions will inform the development of a preventive care model.

## Introduction

Holistic care and prevention of secondary health conditions (SHCs) are unmet needs for people with spinal cord injury (SCI). Living with a chronic health condition, such as a SCI, increases the risk of comorbidities such as non-communicable diseases and SHCs such as pain, pressure sores, and bladder and bowel problems (Jensen et al. 2012; Rimmer, Chen & Hsieh 2011). Secondary health conditions have a significant impact on the individuals’ health (Pilusa, Myezwa & Potterton 2021), work productivity (Callaway et al. 2015), inclusion (Fuseini, Aniteye & Alhassan 2019), and they eventually worsen the primary disability (Richardson et al. 2019). Preventing and managing SHCs is a life-long commitment that requires continued support and interventions to minimise their occurrence.

**FIGURE 1:**
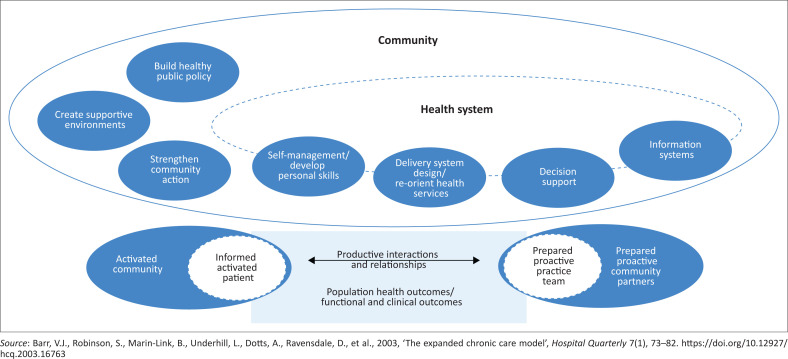
Elements of the expanded chronic care model.

**FIGURE 2:**
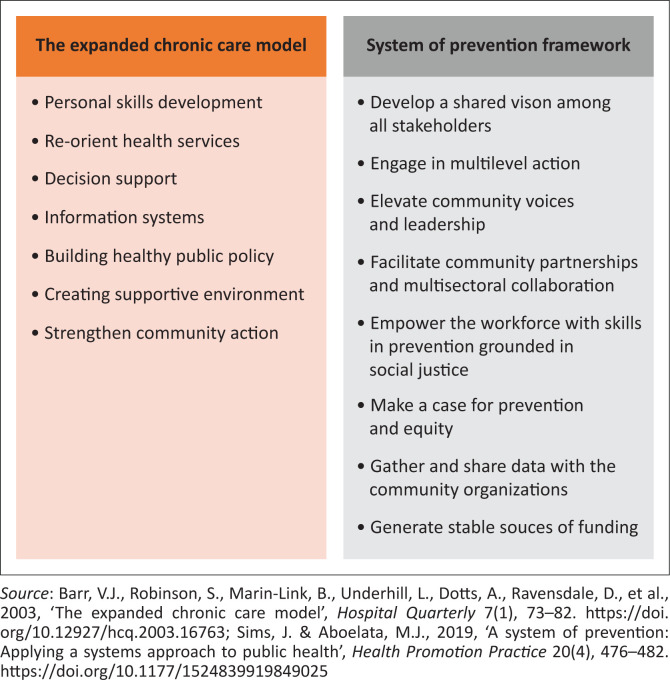
Elements of the expanded chronic care model and the system of prevention framework.

There is a need for a comprehensive care model that can support prevention care for SHCs and address multiple and complex factors which influence prevention care. Evidence on the prevention of SHCs has looked at isolated interventions, which tend to be linearly oriented, assuming that single interventions (e.g. patient education) in isolation will improve health outcomes (Tramonti, Giorgi & Fanali 2020; World Health Organization 2009). Systems thinking approaches to patient care are promoted because they address the whole system, the linkage between the different influencing factors, system behaviours, and encourage multilevel strategies (Atun 2012; World Health Organization 2009). Examples of care models that have incorporated systems thinking to improve health outcomes are the expanded chronic care model (Barr et al. 2003) and the system of prevention framework (Sims & Aboelata 2019). The expanded chronic care model (Figure 1) is a framework used to design interventions for managing chronic diseases, by incorporating population health promotion principles in the health system organisation and community (Barr et al. 2003). The system of prevention is a framework that guides the development of sustainable prevention approaches to produce better health outcomes (Sims & Aboelata 2019). Figure 2 presents the elements from both the expanded chronic care model and the system of prevention framework. Both the expanded chronic care model and the system of prevention framework emphasise the role of health influencing socio-ecological factors, multi-sectoral collaboration and promote multilevel strategies to address complex health problems.

Good health among people with disabilities is a fundamental right central to attaining sustainable development goals (SDG) (United Nations 2006; World Health Organization 2017). To promote health and well-being among people with SCI, we need services and interventions designed to minimise disability and improve access to comprehensive health services. In response to the WHO Rehabilitation 2030 call to action to develop need-based and comprehensive rehabilitation service delivery models (World Health Organization 2017), this study aimed to identify the services and interventions needed to prevent SHCs across the life span of people with SCI. The specified services and interventions can inform the development of a SHCs prevention care model.

## Methodology

### Study design

A descriptive qualitative approach was adopted using focus group discussions. The advantage of using focus group discussion is that discussion and dialogue on ideas specific to the study question can be facilitated.

### Context

The public health system in South Africa is overburdened, serving 84% of the population (Mayosi & Benatar 2014). It is mainly medically oriented, with less focus on rehabilitation care (Morris et al. 2019). An injured person is admitted to a district or tertiary hospital and subsequently referred to the specialised rehabilitation hospital if there is bed space. There are three public specialised rehabilitation hospitals nationally, which results in certain provinces having to transport patients with SCI to regions with a rehabilitation hospital. After discharge, patients with SCI have to travel to the rehabilitation hospital for medical care, consumables and medication because the primary health facilities sometimes lack specific medicine and competence to manage SCI and SHCs (Maart & Jelsma 2014; Sherry 2015). Post-discharge care in the community is minimal due to scarcity of health professionals at primary health clinics, lack of transport and the lower priority placed on rehabilitation care (Morris et al. 2021; Sherry 2015).

### Participants

A purposive sample that includes academics specialising in neurology, public health and social work, a non-profit manager for people with disabilities, rural-based physiotherapists (one runs a disability non-profit organisation), one rehabilitation hospital manager and two provincial rehabilitation managers were recruited.

### Data collection

All the group discussions were conducted online in July 2020 using Microsoft Teams by the primary author and a research assistant. The principal researcher conducted a pilot study with three academics specialising in public health, neurology and spinal cord rehabilitation to clarify the research questions. Pilot study data were included in the analysis because there were no changes to the research question. In the focus groups, the aim of the study was explained to the participants. The participants were asked to respond to the question, ‘Which services and interventions are needed to prevent SHCs throughout the life span of people with SCI?’ All the participants were allowed to ask questions to ensure that everyone understood the research question.

### Data analysis

A qualitative design software, MAXQDA version 2018.2, was used to manage and analyse data. All the group discussions were audio-recorded and transcribed verbatim. Content analysis was conducted whereby the text was divided into meaning units, formulating codes, categorising similar codes, and identifying emerging themes (Erlingsson & Brysiewicz 2017). The first author read the transcripts to gain a general sense of the discussions. The transcripts were coded both inductively and deductively. Codes were grouped into categories and subcategories. A preliminary coding framework was developed and reviewed by all authors. The primary author coded the rest of the transcripts deductively; similar codes were grouped into categories and themes. The other authors reviewed the categories and themes.

### Trustworthiness

Credibility was achieved through audio recording the focus group discussions and holding debriefing sessions with experienced researchers in qualitative research design throughout the research process. For transferability, the study context was described in detail. For dependability, the author kept an audit trail of the research process and explained the methodology in depth.

### Ethical considerations

All participants gave informed consent and permission to record the discussion. The study was approved by the Human Research Ethics Committee of the University of the Witwatersrand (reference number: M170938) and the South African National Health Research Database (reference number: GP201712036).

## Results

The authors conducted four focus group discussions with professionals experienced in rehabilitation. Table 1 presents the participants’ demographic profile.

**TABLE 1:** Demographic profile (*n* = 16).

Variables	Value
Age (years)	45.25 SD 10.14 Min–max 28–61
Gender	4 Male
	12 Female
Level of education	7 MSc
	4 Bachelor degree
	4 PhD
	1 MBChB
Occupation	9 Academic lecturers
	1 Rehabilitation manager (Physiotherapist)
	2 Rehabilitation directors (Occupational therapists)
	1 Rehabilitation medical doctor (medical doctor)
	1 Health consultant and living with SCI
	2 Physiotherapists

SCI, spinal cord injury.

### Section B: Qualitative data analysis results

Four themes emerged from the analysis: patient-centred care, access to resources, promotion of health, and skilled healthcare workers.

#### Patient-centred care

The participants highlighted the importance of patient-centred care in the prevention of SHCs. Care needs to be holistic and must include physical, mental, spiritual, and social aspects of health:

‘What is critical is the issue of participating in excursions, in sports, in a whole lot of activities that would improve the mental and the physical as well as the spiritual well-being.’ (Participant 10)

‘… to prevent isolation which leads more into all these secondary complications … try to find ways of re-integrating people with spinal cord injury back into the community and have them play active roles in society.’ (Participant 13)

The participants also commented on the importance of continuity of care. Patient care needs to be continuous through follow-up care, conducting home visits or seeing patients in the outpatient department to monitor the patients’ health and identify risk factors for SHCs:

‘Different facilities can incorporate follow-up visits, maybe once in three months or once in six months … To monitor, screen and see how the patient is progressing and if there are any risks and secondary complications that are arising later in the rehab process.’ (Participant 14)

‘Follow-up contact … either the person with SCI is coming to the hospital or a home visit so that we detect the emergence of complications early on and intervene before it is too late. We know how pressure sores can accelerate at a very fast pace, so if we pick that up early on, we can then put in intervention measures.’ (Participant 16)

The participants emphasised the importance of collaboration between health professionals from many disciplines in SCI care:

‘The most important thing in spinal cord injuries is the multi-interdisciplinary approach … if no doctor is qualified to talk about bowel and bladder management, then the system falls shut because you cannot expect a physio to take that responsibility.’ (Participant 4)

‘At an institutional level to have a good inpatient care foundation before we can have the patient discharged and going to the community where we have an inter-disciplinary approach with the patient and not have all the health professionals doing isolated prevention interventions.’ (Participant 12)

#### Promotion of health

Promoting health through building personal skills, support systems, and health-promoting policy is vital to the prevention of SHCs, as highlighted by the participants.

**Building personal skill:** In terms of building personal skill, the importance of educating the patients and family on SCI, SHCs, early signs of SHCs and healthy living was expressed by this participant:

‘Educate the patient and the family for early warning signs for incontinence, wheelchair maintenance, what pressure sores look like in the beginning phases before it becomes a pressure sore.’ (Participant 3)

Besides educating on SHCs, health information could include topics on unhealthy lifestyles:

‘So there is the need to educate about obesity, not eating properly, alcohol or substance abuse to cope with the disability.’ (Participant 7).

But the timing for education is essential:

‘Sometimes you want to teach the patients with SCI about prevention when they have not yet accepted the injury, and you find that they don’t remember it five weeks from now.’ (Participant 12)

Other skills necessary for enhancing well-being included self-management and coping skills, as expressed by this participant:

‘Strengthen self-management … so that a person can take care of themselves. Just equipping them with the skills that they need to prevent this complication so that they can do them themselves in case there’s no one around at that time.’ (Participant 13)

‘Empower patients with stress management and coping skills.’ (Participant 7)

**Support system:** Many participants reported how a strong support system through family, peers and the healthcare system helps with SHCs prevention.

‘Looking at the immediate family home … is it conducive for prevention, or does it lead them to have the secondary health conditions? Yes, we might not have the capabilities actually to change that, but once we understand the social standing, we can also include that in the prevention model.’ (Participant 12)

The participants mentioned how peer support through sharing experiences and health information could help people with SCI prevent SHCs:

‘… have peer supporters or peer counsellors that speak to the patients about pressure sore and how long it usually takes to heal just so that they have feedback from somebody who has experienced it.’ (Participant 12)

Lastly, the healthcare system at all levels was identified as a possible source of support when rendering responsive care:

‘Also, ensuring that our referral systems within the health sector are effective. If I’m referring a client, I know who this client is going to, and it doesn’t end there, and I make sure that the client is aware in terms of whom to contact when they get to a certain point in terms of the healthcare system to ensure that we do not have a loss to follow up, which often happens.’ (Participant 8)

**Health-promoting policy:** The participants commented on the importance of policies that enable and support the prevention of SHCs.

‘… government policies must enable the reduction of secondary complications.’ (Participant 1)

The participants also highlighted the need to have minimum standards when it comes to patient care:

‘There is a standard for seating, there’s a good thing for wheelchairs that have come out but the rest of therapy, the rest of rehab, especially with spinal there is no standard protocol in this country, and that is a huge deficit in the service.’ (Participant 4)

#### Access to resources

The participants discussed how access to basic resources such as water, social grants, assistive devices, and medication facilitated the prevention of SHCs.

Lack of clean water hinders self-management practise for individuals with SCI, as expressed by this participant:

‘Access to water and electricity. You can’t expect people to do intermittent catheterization if they don’t even have water.’ (Participant 1)

Additionally, the participants highlighted the importance of accessible medication and consumables in the prevention and management of SHCs:

‘… Ensuring that supplies that go with the management of spinal cord injury at home are available … incontinence products, the whole package must be available to manage urine and waste products.’ (Participant 16)

‘Consistent supply of drugs and continence products is a biggie where in KZN we have battled to have a good supply of Baclofen. … sometimes it’s just been stock out related.’ (Participant 6)

#### Skilled healthcare workers

The participants expressed their views on health professional competence in SCI care and the need for continual training to support prevention care:

‘I also thought of healthcare workers who are trained to do regular home visits.’ (Participant 11)

‘Training of health caregivers about the area of prevention.’ (Participant 13)

## Discussion

This study aimed to identify the services and interventions that are considered essential to prevent SHCs throughout the life span of people with SCI. Four themes emerged from the analysis: patient-centred care, access to resources, promotion of health, and skilled healthcare workers. There has been limited research on the prevention of SHCs in people with SCI.

Our findings showed that a patient-centred care approach is crucial to enhance the prevention of SHCs among people with SCI. Patient-centred care is a long-term and empowering approach to patient care that is needs-oriented, collaborative, inclusive of family and that engages the patient as a critical contributor to personal health (Coulter & Oldham 2016). To enhance patient-centred care, health professionals must respect and listen to the patients’ illness experience, encourage self-care and advocate for patients to make navigating the health system easier (Hudon et al. 2012; Lindberg et al. 2013). Evidence shows that a patient-centred approach improves health outcomes, patient activation levels, and adherence to treatment (Kuipers, Cramm & Nieboer 2019). A positive work environment and professional leadership are essential to facilitate patient-centred care practice (Jardien-Baboo et al. 2016; Poitras et al. 2018). Secondly, continuous health professionals training is needed to ensure competent health professionals who are proactive to prevent diseases (Jardien-Baboo et al. 2016).

There is a need for continual health professional training to support prevention care. The lack of knowledge on SCI and SHCs care among health professionals disempowers people with SCI and leaves them desperate for care (Fuseini, Aniteye & Kofi-Helegbe 2018; Guilcher et al. 2013; Zanini et al. 2020). Because prevention care relies on the partnership between health professionals and persons with SCI, health professionals need knowledge on SCI, SHCs, biopsychosocial approach to care, preventive care and soft skills such as communication, negotiating, goal setting and interdisciplinary teamwork skills (Tramonti et al. 2020; Zanini et al. 2019). Training health professionals on the topics mentioned above should be part of the undergraduate curriculum and continuous in-service training.

The promotion of health maintenance is critical in SCI care to minimise the occurrence of SHCs. The study’s participants highlighted the importance of promoting health by building personal skills, support systems, and health-promoting policy. People with SCI can be empowered with SCI and SHCs prevention care information (Chang et al. 2017; Van Loo et al. 2010) and be supported to live healthily. The support system can be from family, friends and health professionals (Lindberg et al. 2013). Involving the family and peers in rehabilitation and prevention care post-discharge can bridge the care needs gap by ensuring patient care continuity (Sherry 2015). Lastly, health professionals can be part of the support system for people with SCI at all levels of care through follow-up care, regular home visits, outreach services, and telehealth interventions (Dejong & Groah 2015; Sherry 2015).

Health-promoting policies are needed to enable and support the prevention of SHCs. In the absence of a disability policy, South Africa developed and adopted the Framework and Strategy for Disability and Rehabilitation services 2015–2020 (South African Department of Health 2015). The framework outlines disability management through accessible rehabilitation services, which include disease prevention and health promotion. To date, there is no published outcomes or impact of the Framework and Strategy for Disability and Rehabilitation services 2015–2020. South Africa’s challenge is not the absence of health-promoting policies for people with disabilities, but the poor policy implementation and the low priority placed on rehabilitation care for people with long-term care needs (Morris et al. 2019; Sherry 2015). Unless there is a shift from curative care to a biopsychosocial focus to care and to prioritise rehabilitation, health promotion, and disease prevention for people with disabilities, health outcomes will not improve.

The last theme that emerged was access to resources that can help prevent and manage SHCs. The participants highlighted the importance of accessible medication and consumables in the prevention and management of SHCs. Pharmaceutical care is necessary for some of the SHCs, such as pain, spasm, and bladder and bowel management (Patel, Milligan & Lee 2017). For example, assistive devices are essential for mobility, self-care and to facilitate participation in life activities. The shortage of medical resources needed to prevent SHCs can worsen health outcomes and increase economic vulnerability due to out-of-pocket expenses for assistive devices and over-the-counter medication purchases (Hanass-Hancock et al. 2017). To safely manage bladder and bowel problems, clean water is necessary. However, due to unemployment and social inequalities, access to essential services such as safe water and electricity can be unaffordable (Hanass-Hancock et al. 2017; Statistics South Africa 2014; The World Bank 2018). This study points to the importance of intersectoral collaboration when planning holistic care for people with SCI, thus ensuring access to essential services, consumables, and pharmaceutical products.

**FIGURE 3:**
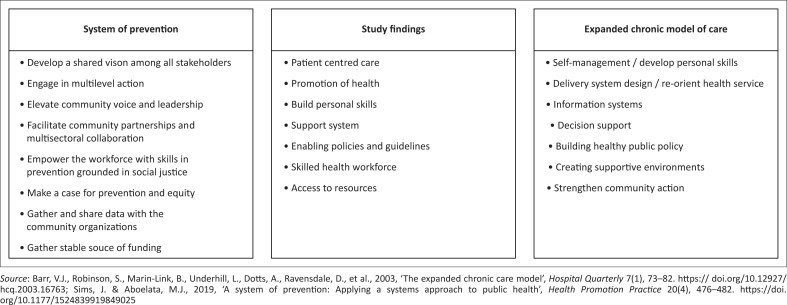
Comparison between the emerged themes and the elements from the expanded chronic care model and the system of prevention framework.

The study findings were comparable to the expanded chronic care model and system of prevention framework (Figure 3) (Barr et al. 2003; Sims &Aboelata 2019). The elements that were missing in the study findings were information system, gathering and sharing data with community organisations, community partnership and multi-sectoral partnership. Research on disability needs can help build a case for prevention care intervention and equity. However, research should include people with disability and relevant stakeholders in the co-production of knowledge and implementing interventions (Redman et al. 2021). Co-production of knowledge through participatory action research can foster mutual learning, empower the community, and facilitate the translation of research into practice. Building partnerships with the community and other sectors is central to sustainable development and it recognises the vital role other sectors play in shaping health outcomes. A collective understanding and buy-in from all sectors can inform planning, practices, and processes when redesigning systems to achieve better health outcomes.

## Conclusion

To strengthen SHCs prevention care among people with SCI, the prevention model of care needs to include services and interventions that enhance patient-centred care, ensure access to resources and, promote health by skilled healthcare workers. The interventions and services target different levels and stakeholders, namely, the patients, health professionals, health system care approach and resources from the health system and outside the health system. Systems perspective in service delivery and development of interventions promotes a broader understanding of the complexities of health problems and informs intervention design (Atun 2012; MacLachlan & Scherer 2018).

## Limitation

This study has some limitations due to the coronavirus disease 2019 (COVID-19) pandemic. Firstly, the focus group discussions were conducted online, and we had to limit the discussion time. Secondly, participants with SCI could not take part in the study due to inadequate access to technology. Although therapists based at a rehabilitation hospital were invited to participate, none accepted the opportunity to participate in the discussion, possibly due to the healthcare challenges from the COVID-19 pandemic. Future research must seek the opinion of people with SCI and therapists. Thirdly, future research should include the biokineticists because they are also important members of the multi-disciplinary team managing SCI patients.

## Implications

Research: Future research can use systems thinking tools such as concept mapping, causal loop diagram, or modelling to conceptualise the proposed solutions and model their outcomes.

Practice: Health professionals are encouraged to empower people with SCI with knowledge on SHCs, and self-management skills.

Education: Health professionals need continuous in-service training on patient-centred care and the importance of preventive care.
